# Evaluating changes in workplace culture: Effectiveness of a caregiver-friendly workplace program in a public post-secondary educational institution

**DOI:** 10.1371/journal.pone.0250978

**Published:** 2021-05-14

**Authors:** Anastassios Dardas, Allison Williams, Li Wang

**Affiliations:** 1 School of Earth, Environment & Society, McMaster University, Hamilton, Ontario, Canada; 2 Offord Center for Child Health Study, McMaster University, Hamilton, Ontario, Canada; Xiamen University, CHINA

## Abstract

**Background:**

Workplace experience, defined as the evaluation of the work environment and performance, and a characteristic of workplace culture, can influence an employee’s work-life balance. Most carer-employees, who combine paid full-time work and informal caregiving responsibilities, struggle to maintain a healthy work-life balance. Caregiver-Friendly Workplace Programs are designed to improve the work experience, and ultimately, the work-life balance of carer-employees. The purpose of this study is to identify changes in workplace culture through the examination of the efficacy of a caregiver-friendly workplace program on workplace experience. First, we identify whether awareness of a caregiver-friendly workplace program directly increases the amount of work support received and, in turn, improves workplace experience. Second, we will examine if significant differences in the amount of work support received translates into an improved workplace experience for carer-employees over time.

**Methods:**

Two university-wide online surveys were conducted separately; time 1 (T1) during the summer of 2015, and time 2 (T2), in the summer of 2017. In each survey, nearly 7000 employees received the invitation to participate with a response rate ranging 10% (T1) to 12% (T2). Respondents were asked about their sociodemographic characteristics, caregiving responsibilities (if applicable), awareness of caregiver-friendly workplace program, types of work support received, and work experience. Reliability analyses was conducted for three scales: *awareness of caregiver-friendly workplace program; work support*, and; *workplace experience*. Proportional T-tests were used to examine the difference amongst the intervention scales over time. Structural equation modeling (SEM), via path analysis, was used to investigate the causal indirect (*awareness of caregiver-friendly workplace program* to *work support* to *workplace experience)* relationship that define the workplace culture.

**Results:**

No significant changes in workplace culture were found over time. However, awareness of caregiver-friendly workplace programs is shown to positively impact the *amount of support received*, which sequentially improves *workplace experience*, and ultimately *workplace culture*. This therefore suggests that the implementation of caregiver-friendly workplace programs is potentially effective.

**Conclusions:**

Results suggest that *amount of support received*, and *workplace experience* would be better reassessed via a longer time period (i.e., 5 yr. window), and improved support for managers and supervisors is needed to supplement relationships with their employees.

## Introduction

Due to the combination of increased life expectancy and low fertility rates, the global population is ageing; the aged population was 962 million in 2017 and is projected to be 2.1 billion in 2050 [1]. Some countries (i.e., Japan and Italy) are already experiencing dramatic changes in labour force participation, such as the potential support ratio for seniors (age 65+). Japan has a ratio of 2.1 people available to take care of the older population, in contrast to Canada at 3.8, and the United States at 9 [2]. Since 2016, Canada’s seniors are outnumbering youth (under 15 yrs.); this is the first time such an occurrence has happened in Canadian history [3]. These trends are not only impacting economies, but are also having affects on the labour force, as more employees are expected to not only work for pay, but also provide informal, unpaid care to family, friends, and neighbours in their lives. Carer-employees are defined as individuals that provide unpaid care to a family member or friend while working in paid employment [4,5]. The term is sometimes referred as ‘employed carers’ or ‘carer workers’ and does not include home health care professionals or care-related professions (i.e., nurses and physiotherapists). Currently, there are 6.1 million carer-employees in Canada, and the majority are the most experienced employees given their age cohort (45–64) [4]. A lack of workplace support can lead to dire health and financial consequences for carer-employees. Canada loses $5.5 billion annually due to work absenteeism and counter-productivity that result from caregiving responsibilities [4,6]. In the United States, this is estimated to be $74 billion of annual loses [7]. Such losses are often caused by a conservative workplace culture. The purpose of this study is to identify changes in workplace culture through the examination of the efficacy of a caregiver-friendly workplace program on workplace experience in a public, post-secondary public institution in Canada.

To have a better understanding of the efficacy of caregiver-friendly workplace programs, the literature review will overview: 1) the detrimental effects caregiver burden has on employers, and 2) the importance of caregiver-friendly workplace programs.

## Literature review

### Consequences of caregiver burden

Due to the combination of demographic changes, rising cost of living, and changes in household roles, the workplace has experienced dramatic changes. These changes include: 1) employees prolonging their stay in the workforce, thus delaying retirement; 2) more women are represented the workforce due to the cost of living and the need for dual-income families [8], and; 3) more employees are simultaneously providing unpaid, informal caregiving, classified as carer-employees. Thirty-five percent (35%) of the Canadian labour force are carer-employees, of which 45% are experiencing work-life balance struggles due to care responsibilities, with many experiencing caregiver burden [5]. Forty-five percent (45%) of these carer-employees are experiencing a range of consequences, including taking temporarily leave from work, reducing work hours, counter-productivity, and even resigning [5]. This results in negative economic impacts (as mentioned earlier), and recruitment and retention issues. If employers are proactive, they recognize the problem and work to improve the work-life balance of their employees. One approach to improving the work-life balance of employees, and especially carer-employees, is the implementation of carer-friendly workplace programs.

### Caregiver-friendly workplace program & workplace experience

Fortunately, progressive employers are working to retain talent, and mitigating counter-productivity and absenteeism through the implementation of caregiver-friendly workplace programs, such as the Compassionate Care Benefit (CCB), compressed work weeks, flextime, and prolonged leaves of absence. Caregiver-friendly workplace programs are implemented to optimize the workplace experience, which translates into an improved work-life balance for employees and especially carer-employees. Several studies have highlighted the following benefits of implementing caregiver-friendly workplace programs: 1) increased employee retention; 2) less employee turnover; 3) reduced absenteeism; 4) optimistic staff morale; 5) enhanced employee satisfaction; 6) continuous development of employee skills and knowledge, and; 7) excellent company reputation [9–14]. All these positive traits reflect the workplace culture through a positive workplace experience for employees. Further, they require the support and involvement of senior management (i.e. CEOs, senior managers, and human resources) as well as direct communication between supervisors and employees [15–17].

In the context of carer-employees, a progressive workplace culture translates into positive workplace experience. A progressive workplace culture is characterized as: 1) recognizing the integration between work and family life as a social responsibility through implementing caregiver-friendly workplace programs, and; 2) having leadership in the workplace (CEOs, senior managers, human resource personnel, and/or supervisors/managers) implement and bring awareness to caregiver-friendly workplace programs. With respect to the first criteria, caregiver-friendly workplace programs provide flexibility, trust, and open communication [18], and are recognized as an economic benefit for companies [18]. While this illustrates progress, many employers still struggle to implement caregiver-friendly workplace programs due to either: the nature of the job (i.e. manufacturing, emergency personnel); the immediate direct costs; lack of awareness and knowledge, and/or; lack of support from senior management [18]. What may be more disappointing are employers who implement caregiver-friendly workplace programs to display good public reputation, yet internally do not support them. Ultimately, the amount of support for caregiver-friendly workplace programs that comes from senior management reflects the carer-employees’ workplace experience. HR representatives are often the first to be approached with respect to caregiver-friendly workplace programs [19]. If the support and transparency from senior management (particularly HR) exists, then employees are generally more aware of caregiver-friendly workplace programs [19].The next section introduces workplace culture as a conceptual framework to examine the efficacy of caregiver-friendly workplace programs on workplace experience.

### Workplace culture framework

**Fig 1:**
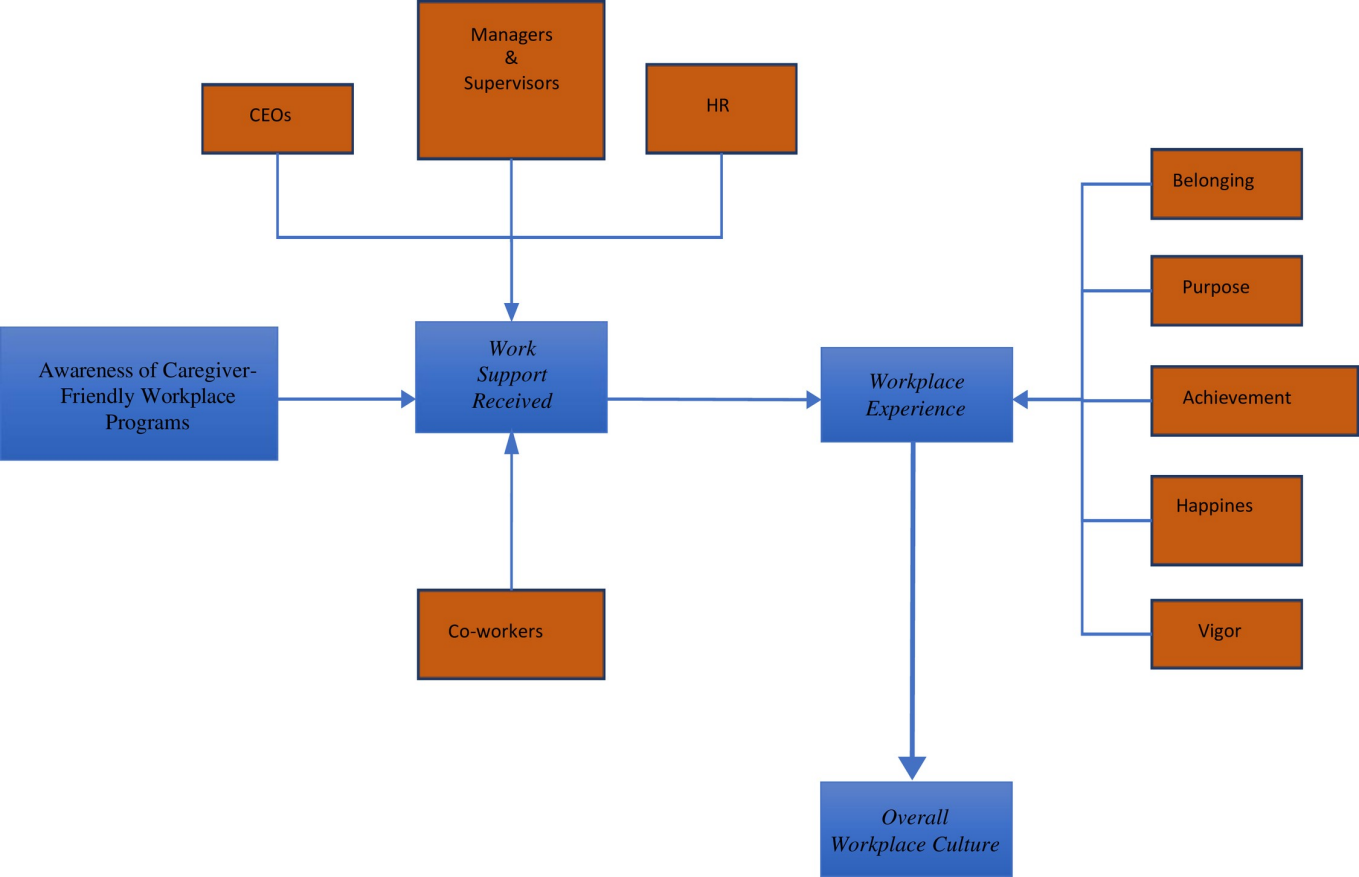
Conceptual framework.

The Smarter Workforce Institute at IBM conducted a major research study to measure employee experience, including the development of an index and framework. By their definition, employee experience is a list of feedback characteristics that describes the employee’s experience at work [20]. These feedback characteristics internally sum up the employee experience index, which consists of a sense of belonging, purpose, achievement, happiness, and vigor [20]. External factors that impact workplace experience are the behaviours and actions from leadership and management, which translates into workplace practices and work-life balance. Workplace practices and work-life balance affect the employee experience, reflecting the overall workplace culture. This path describes IBM’s framework (work support to employee experience) on employee experience. Our conceptual framework on workplace culture is a hybrid derivation of IBM’s framework, with the main borrowed from the caregiver-friendly workplace program literature [16,18,19]. Fig 1 broadly displays the workplace experience framework for our study.

Past studies have shown that leadership behaviours, such as support and relationship quality amongst employees impacts employees’ wellbeing and job satisfaction [21–25]. In the framework noted above, the combination of *awareness of caregiver-friendly workplace programs (CFWPs)* and *work support received* define the type of behaviour and action from leaders, whereas *workplace experience* defines the employees’ wellbeing and overall job satisfaction. This conceptual framework constructs the overall workplace culture; however, it has not been used to investigate the efficacy of caregiver-friendly workplace programs over time in a post-secondary public institution in Canada. Therefore, this study is to address the following research objectives:

1. Does awareness of CFWPs directly increase the amount of support received by employees? If so, does the amount of support received improve workplace experience?
2. Has there been a positive change in workplace culture over time, based on significant differences in amount of support received, as reflected in improved workplace experience for carer-employees?

In general, this research contributes to the caregiving literature, as well as the human resource/management literature through identifying whether awareness of CFWPs in a Canadian workplace improves workplace culture. The idea of improving workplace culture is to enhance carer-employee’s work-life balance.

## Materials and methods

As discussed elsewhere, the larger research project has three phases [26]. Phase A determined the effectiveness of caregiver-friendly workplace program to identify impacts on carer-employee health. As an extension of Phase A, Phase B implemented a cost-benefit and cost-effectiveness analyses to assess the economic outcomes of caregiver-friendly workplace program. Separate from Phase A and Phase B, but part of the same project, Phase C (this paper) employed an implementation analysis to determine the effectiveness of CFWPs with respect to changes in workplace culture over time, via the analysis of the characteristics of support and workplace experience. Cross-sectional data for Phase C was collected from a survey at two different time periods. The Research Ethics Board at McMaster University reviewed and provided ethics exemption for this study (approval #2016 068). The surveys were hosted online through the LimeSurvey platform and consisted of five themes: 1) sociodemographic characteristics; 2) caregiving responsibilities (if applicable); 3) awareness of caregiver-friendly workplace programs; 4) amount of work support received, and; 5) workplace experience. Table 1 lists the manifest items for the scales created: (1) *awareness of caregiver-friendly workplace programs*; (2) *amount of work support received*, and; (3) *workplace experience*.

**Table 1:** Scales & sociodemographic statistics across time.

Variable	Value	T1 (n = 747)	T2 (n = 816)	Significance
**Awareness**				
AW1 –Employee Family Assistance Plan	Yes	53.3%	65.9%	***
AW2 – 3^rd^ Party Service (Counseling)	Yes	41.7%	51.8%	***
AW3 – 3^rd^ Party Service (Specialist)	Yes	11.1%	18.6%	***
AW4 – 3^rd^ Party Service (Online/Presentation)	Yes	11.8%	21.9%	***
AW5 –Flexible Work	Yes	48.3%	47.0%	
AW6 –Personal Leave Days	Yes	82.1%	81.4%	
AW7 –Bereavement Leave	Yes	72.9%	68.4%	*
AW8 –Compassionate Care Benefit (CCB)	Yes	13.2%	14.8%	
**Support**				
S1 –Co-workers	Yes	69.6%	69.6%	
S2 –Supervisor	Yes	62.4%	62.0%	
S3 –Human Resources	Yes	32.4%	35.9%	
S4 –Family	Yes	89.4%	88.9%	
S5 –Friends	Yes	84.1%	83.2%	
**Workplace Experience**				
WX1 –I am satisfied with the amount of involvement I have in decisions that affect my work.	Strongly Agree	29.7%	27.7%	
	Agree	48.2%	52.0%	
	Disagree	16.5%	15.1%	
	Strongly Disagree	5.7%	5.2%	
WX2 –I feel I am well rewarded (in terms of praise and recognition) for the level of effort I put out for my job.	Strongly Agree	28.9%	25.9%	
	Agree	38.5%	45.3%	**
	Disagree	24.6%	21.8%	
	Strongly Disagree	7.9%	6.9%	
WX3 –In the last 6 months, too much time pressure at work has caused me to worry, “nerves” or stress.	Strongly Agree	20.2%	24.3%	.
	Agree	33.9%	35.9%	
	Disagree	34.9%	32.2%	
	Strongly Disagree	10.9%	7.6%	*
WX4 –In the last 6 months, I have experienced no worry, “nerves” or stress from mental fatigue at work.	Strongly Agree	6.8%	7.5%	
	Agree	22.9%	20.0%	
	Disagree	42.6%	45.6%	
	Strongly Disagree	27.7%	27.0%	
WX5 –I am satisfied with the fairness and respect I receive on the job.	Strongly Agree	26.9%	25.4%	
	Agree	50.2%	51.7%	
	Disagree	15.7%	17.2%	
	Strongly Disagree	7.2%	5.8%	*
WX6 –My supervisor supports me in getting my work done.	Strongly Agree	37.5%	36.4%	
	Agree	47.3%	48.7%	
	Disagree	10.6%	10.9%	
	Strongly Disagree	4.7%	4.0%	
**Demographics**				
Age	45 yrs. or less	44.5%	37.3%	**
	46+ yrs.	55.4%	62.7%	*
Gender of Respondent	Woman	82.9%	80.6%	
	Man	17.1%	19.4%	
Marital Status	Single, Widowed, Divorced	31.7%	25.1%	**
	Married	68.3%	74.9%	**
Highest Level of Formal Education Completed	Trades Certificate or Less	8.4%	5.4%	*
	College/GCEP and above	91.6%	94.6%	*

Significance = p < 10% (0.1)

* = p < 5% (0.05)

** = p < 1% (0.01)

*** = p < 0.1% (0.001).

*Awareness of caregiver-friendly workplace programs* (AW1-AW8) is defined as whether the employee (regardless of he/she is a carer-employee or not) is aware of the programs that would improve their work-life balance, such as an employee family assistance plan or bereavement plan. *Amount of support received* (S1-S5) incorporates work-related and non-work-related supports, such as that provided by the employee’s supervisor and/or family member, respectively. *Workplace experience* (WX1-WX6) asks several questions pertaining to workplace and job satisfaction, and work-related mental stress.

Access links to the surveys were sent out to all employees (n = 7000) at two different time periods through the Human Resources Department email listserv: Time 1 (T1) was implemented in summer of 2015; and Time 2 (T2) was implemented in the summer of 2017. Based on the total number of employees working at the post-secondary public institution, the response rate for T1 was 10.9% (n = 761) and T2 at 13.5% (n = 948). After removing “NA” values and incomplete responses, the final observation count for T1 was at 747 and T2 at 816. While there is no evident reason as to why the response rate was low, one possibility may be due to the timing of sending out the surveys. The summer season is generally a slow period of engagement as many employees are on vacation.

As these surveys were collected at a six-month time interval, we hypothesized that an increased *awareness of caregiver-friendly workplace programs* over time would correspond to an increase in the *amount of work support received*, and ultimately improve *workplace experience*. Hence, a positive feedback loop would reflect the implementation of caregiver-friendly workplace programs as effective in improving workplace experience, and ultimately enhance workplace culture. Furthermore, this study expected to see significant improvements in all the scales (awareness, support, and workplace experience) over time.

The three scales used “Yes/No” questions, as well as Likert scale questions. These scales were then converted to numerical values. Cronbach’s alpha was used to measure the internal reliability of the following three scales: 1) *awareness;* 2) *support*, and; 3) *workplace experience*. Descriptive statistics are presented in Table 1 and include the three scales and relevant sociodemographic characteristics at T1 and T2. Age, marital status, and gender were the only sociodemographic items factored for in the analysis, given that they are known to impact the outcome of workplace experience [27–32]. For instance, women may have a lower workplace experience than men due to gender-based barriers, which limits access to information channels [33–35]. Other sociodemographic and caregiving characteristics were not used in the analysis but are presented in Appendix A for reference. Values containing “Other” in the selected sociodemographic variables were dropped due to low numbers.

**Fig 2:**
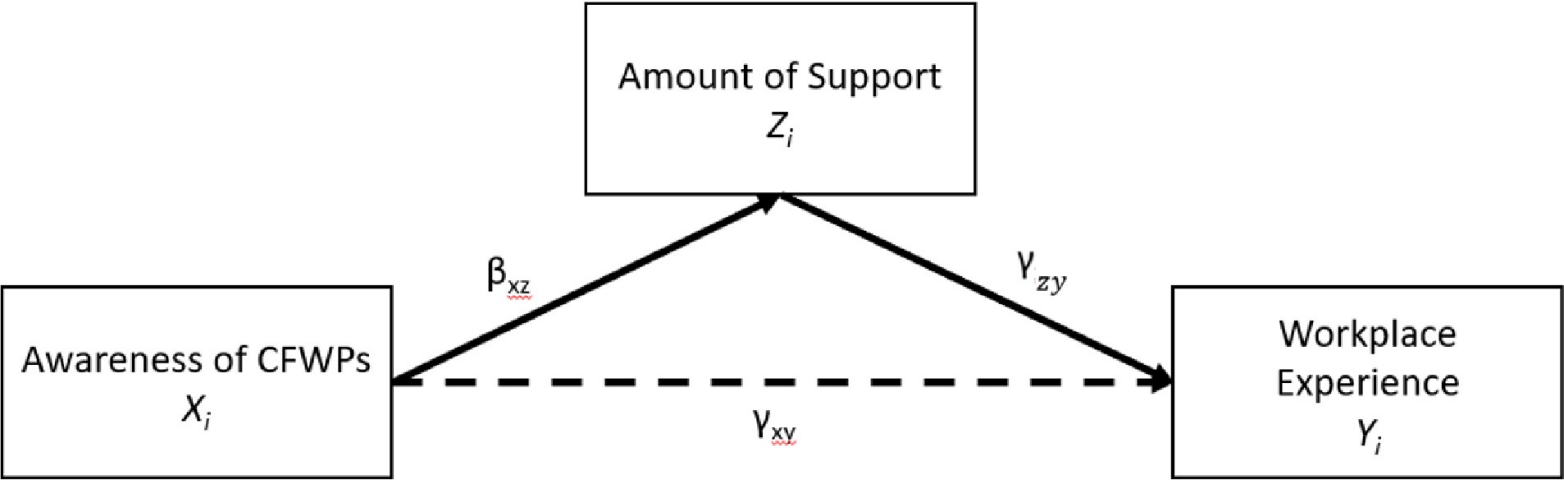
SEM path model.

Guided by the conceptual model, we applied Structural Equation Modeling (SEM) to explore the association between the *awareness*, *support*, and *workplace experience*. SEM is one of the methods that can analyze structural relationships, combining factor analysis and multiple regression analysis for path analyses [36]. Path analysis is a causal modeling version of SEM that examines the relationships between one or more independent variables. The independent and dependent variable are expressed as latent, which are factors that express observed covariation in response [37]. In this context, *awareness of caregiver-friendly workplace programs* is the main independent latent variable. It consists of only the 3rd party services (AW2 –AW4) as these are the ones least likely to be used or acknowledged. AW1 and AW5-AW8 are all standard federal programs. *Amount of support received* is the path latent variable (both dependent and independent). *Workplace experience* was set as the primary latent dependent variable. It composes approximately all the workplace experience items as they measure job satisfaction. To see changes in the *amount of support received* and *workplace experience* over time the variable time, was factored in as an independent variable. Fig 2 shows a path diagram for the path analysis between the three latent variables: awareness of CFWPs (*x_i_*), amount of support received (*z_i_*), and workplace experience (*γ_i_*).

The SEM equation is displayed below:

$$z_{i}=\beta_{0}+\beta_{xz}x_{i}$$

$$\gamma_{i}=\gamma_{0}+\gamma_{zy}z_{i}+\gamma_{xy}x_{i}$$

The direct effect is the direct pathway (*γ_xy_*) from awareness of CFWPs to workplace experience. The indirect effect delineates the pathway from awareness of CFWPs to workplace experience through the mediator variable (amount of support received). This mediated path constitutes through the product of *β_xz_* and *γ_zy_*. Lastly, the total effect accounts the sum of the direct and indirect effects of awareness of CFWPs on workplace experience, *γ_zy_*+*β_xz_γ_zy_*. All the coefficients in the latent variables were standardized and used for multiple regression analyses. Three commonly used model fit indices were assessed utilizing the comparative fit index (CFI), root mean-square error of approximation (RMSEA), and standardized root mean square residual (SRMR) to examine the quality of the SEM model [38]. For the CFI and Tucker Lewis Index (TLI), a value of 0.90 or higher is considered acceptable fit, with those closer to 0.95 considered to be a well-fitting model [38,39]. For RMSEA, a fit of less than 0.08 and, for SRMR, a fit of 0.08 or less indicates a good fit [38,39]. All analyses were conducted in RStudio (v. 3.4.4), a statistical integrated development environment (IDE) program for R.

## Results

### Descriptive statistics

Table 1 records the sociodemographic characteristics and the three scales that were used in the path analysis models. Additional sociodemographic and caregiving characteristics can be viewed in Appendix A. On each time surveys most participants were aged 46 years of age or older. As expected, most of survey respondents were female, married, and having obtained at least a college degree or above as the highest formal education received. Descriptions of the three scales are subdivided into three short paragraphs below.

### Awareness of caregiver-friendly workplace programs scale

Most respondents in both time periods were aware of personal leave days (over 80%), whereas few recognized the Compassionate Care Benefit (13–15%) and the availability of 3rd party online/presentation services and specialists (11–22%). Fortunately, all 3rd party services, as well as the Employee Family Assistance Plan, had a significant increase in awareness over time. The Bereavement Plan was the only caregiver-friendly workplace program to have a significant decrease in awareness over time.

### Amount of support received scale

For the *amount of support received* scale, the most supportive groups towards respondents were family (~89%), followed by friends (~84%) and co-workers (~69%). Human resources (32–36%) are perceived to be the least supportive group.

### Workplace experience scale

For the *workplace experience* scale, most respondents were satisfied (accounts “strongly agree” and “agree” values) with: the amount of involvement they have in decisions that affect their work (78–80%); feeling well rewarded for the level of effort (67–71%); supervisor support (~85%), and; the fairness and respect they receive when on the job (77%). The latter seemed to have a significant decrease in “strongly disagree” over time. On the negative side, most respondents experienced too much time pressure at work. Fortunately, there has been a significant decrease in “strongly disagree” over time. While there have not been many changes over time, the overall workplace experience has been positive.

### Reliability analysis

Cronbach’s alpha reported all scales for both time periods to be reliable for analyses. The scores for each scale from T1 to T2 respectively is as follows: *awareness* (8 items) from 0.67 to 0.72; *support* (5 items) from 0.67 to 0.68, and *workplace experience* (6 items) from 0.80 to 0.83 (Table 2). This indicates the internal consistency of both *awareness* and *support* as acceptable, and *workplace experience* as good [40,41].

**Table 2:** Cronbach’s alpha.

Time	Theme	Cronbach’s Alpha
T1	Awareness	0.67
	Support	0.67
	Workplace Experience	0.80
T2	Awareness	0.72
	Support	0.68
	Workplace Experience	0.83

### Structural equation modeling

Results from the path analysis addressed the first research objective. The relationships amongst the three latent variables correspond to the path of the conceptual framework (Fig 1). Generally, work supports (i.e., HR, supervisors) that are more aware of caregiver-friendly workplace programs tend to be more supportive towards their employees. Results show employees to have a better *workplace experience* and thus, a positive workplace culture. More specifically, while controlling for age, sex, and marital status, latent regression shows an increase in *awareness*, and being married augments the *amount of support received*. In turn, the augmented *amount of support received* ultimately improves the *workplace experience*.

Under the modification indices section (Table 3), the group SEM model reported excellent goodness of fit for both, the CFI (0.968) and TLI (0.96), as well as the RMSEA (0.04) scores. Path analysis resulted in a positive association between *awareness* and *support*, and *support* and *workplace experience* (Z = -1.73; p = 0.042) did not highlight any significant differences over time.

**Table 3:** Grouped SEM path analysis of workplace culture across time.

Latent Variable	Standardized Coefficient (SE) p < 10%; * = p < 5%; ** = p < 1%; *** = p < 0.1%	Std. Error	P-Value
*Awareness*			
AW2	0.282***	0.013	0.000
AW3	0.272***	0.010	0.000
AW4	0.303***	0.010	0.000
*Support*			
S1	0.152***	0.008	0.000
S2	0.294***	0.009	0.000
S3	0.157***	0.008	0.000
*Workplace Experience*			
WX1	0.457***	0.016	0.000
WX2	0.540***	0.018	0.000
WX4	0.186***	0.017	0.000
WX5	0.524***	0.016	0.000
WX6	0.448***	0.015	0.000
*Regression*			
*Support ~*			
Awareness (a)	0.061^.^	0.032	0.054
Marital (Ref: Not Married)	0.185***	0.063	0.004
Age (Ref: 46+)	0.063	0.058	0.279
Gender (Ref: Male)	0.007	0.073	0.926
Time (Ref: Time 1)	0.026	0.057	0.642
*Workplace Experience ~*			
Support (b)	0.965***	0.055	0.000
Marital (Ref: Not Married)	0.101	0.068	0.137
Age (Ref: 46+)	0.042	0.062	0.500
Gender (Ref: Male)	0.022	0.078	0.779
Time (Ref: Time 1)	-0.027	0.061	0.653
Awareness (c)	-0.058^.^	0.034	0.092
*Effects*			
Indirect	0.059^.^	0.031	0.055
Total	0.001	0.042	0.975

Modification indices of the grouped model are significant with: CFI (0.968); TLI (0.960); RMSEA (0.042); SRMR (0.034).

## Discussion

The main purpose of this study was to examine the effectiveness of caregiver-friendly workplace programs by exploring the path relationship between *awareness of caregiver-friendly workplace programs* and *workplace experience*. More specifically, we examined the workplace culture via *workplace experience* over time. Although the purpose of caregiver-friendly workplace programs is to improve the work-life balance of carer-employees, it also impacts the non-carer-employees labour forces’ *workplace experience*. The university-wide surveys at a Canadian post-secondary institution for T1 and T2 allow us to examine: *awareness of caregiver-friendly workplace programs*; *amount of support received*, and; *workplace experience* over time. Respondents from both surveys were: less aware of third-party caregiver-friendly workplace program services; uncertain about receiving support from their human resource departments, and; feeling stress and time pressure at work. However, most respondents were found to have a positive workplace experience, which translates to positive workplace culture. This could be due to the perks of working in the academic sector, such as having more vacation days and work flexibility than in the corporate/business sector. From the descriptive statistics results, there were significant changes over time across the awareness latent scale, except for flexible work, personal leave days, and Compassionate Care Benefit. Most of the significant changes in awareness were positive and relate to third party services. Despite having an overall positive workplace experience in both time periods, due to supervisory support (WX6), satisfaction with work (WX1), and feeling well rewarded (WX2), there were no significant changes in support and by extension, workplace experience. The coefficients from the SEM model somewhat reflect this statement. The SEM model demonstrated that an increase in awareness of caregiver-friendly workplace programs improves the amount of support received, which then augments the workplace experience. This is also conveyed with statistical significance from the indirect effect. An employee’s workplace experience relies on the current support of their senior managers and leaders. Generally, the perspectives of workplace experience amongst senior managers and leaders play a critical role [42,43]. Thereby, it is up to senior managers and leaders to decide whether they are willing to acknowledge the work-life balance struggle of their employees (if any), recognize existing programs of support, and allocate them accordingly. Programs are only effectual if senior managers and leaders are proactive in accommodating the needs of their employees. Nonetheless, the SEM model results addresses the first research objective, confirms our hypothesis, and provides a path framework that may be distributed as an essential workplace tool for higher management and decision-makers. Decision-makers can use the path framework to identify and dissolve different types of barriers that prevent the improvements of workplace experience. Different types of barriers may include the age and marital status of employees, as our results have shown. While these sociodemographic actors are beyond the control for employers, they may facilitate employers to cast specific programs (i.e. supportive programs for the young and single) tailored to the needs of their employees.

For *amount of support received*, we did not hypothesize that older or younger workers would have better support, as there are mixed interpretations between the two cohorts. In this case, even if the coefficient was insignificant, younger workers may receive more support in their workplace environment due to their relative inexperience. The literature suggests that older workers tend to have more work experience, stronger work relationships and be more motivated by causes such as community missions, and be more capable of problem-solving without drama [44–46]. Additionally, older workers generally obtain high-level leadership roles and do well in organization, listening, writing skills, and detailed-oriented tasks. These characteristics may explain why older workers are receiving better support. Being married seems to be significant. Married respondents have a higher amount of support, which could translate into having a better workplace experience when compared to single, divorced, or widowed respondents. Overall, awareness of caregiver-friendly workplace programs appears to have indirectly resulted in positive workplace experience. This validates that implementation of caregiver-friendly workplace programs are effective.

To fulfil the second research objective, the time variable was applied to identify any improvements. The time coefficient for support is positive and not significant, indicating an improvement over time. For workplace experience, the time coefficient was negative and not significant, indicating a decrease over time. Thus, there does not appear to be much change in workplace culture over time. Additionally, the direct effect demonstrated a decrease in workplace experience. Both of these do not necessarily indicate that the implementation of caregiver-friendly workplace programs is ineffective or saturated, but rather reflects the workplace experience of the post-secondary institution, while suggesting further improvement. This statement is supported based on the results of the total effect, which had a coefficient hardly above zero. To further improve the workplace experience, we first need to understand what constitutes it. Next, we need to acknowledge the status of the current workplace experience and make suggestions to improve it. In this context, the workplace experience is defined by the amount of support employees received from senior management, reflected in the awareness and level of transparency of caregiver-friendly workplace programs. Situated within the descriptive statistics and the SEM model, the post-secondary institution has an overall positive workplace experience yet may require improvements. Improvements in the workplace experience will require at least one of the following: 1) improve trusting relationships between human resources and employees, as evident in the descriptive statistics and highlighted by Vuksan et al. [19], and; 2) passage of more time for the cultural change needed specific to the knowledge about the caregiver-friendly workplace programs, which takes time to trickle through the network of stakeholders in the workplace [47]. As part of the support group, human resources have access to opportunities and are key advocates in making changes in the workplace environment. More importantly, having a better supportive relationship with employees may improve the workplace experience by establishing trust. This may apply to supervisors, managers, and other senior stakeholders as well. Lastly, the implementation of caregiver-friendly workplace programs will require the passage of time to improve the workplace experience. As evident from the results, two years is not sufficing to make any major improvements in the workplace experience, nor to change the workplace culture and to establish trust. This has been confirmed in other research [48].

### Limitations & future research

There are a couple of limitations to this study that, if addressed, may improve future research. The first limitation is that T2 likely does not have the same respondents as T1 and, if there were, the data from T1 and T2 cannot be connected. As a result, the dataset is not a longitudinal, which makes it difficult to perform a test-retest reliability analysis. If both surveys were to capture the same respondents with a pseudo-identity number, the analytic methods would be different, and the results may be different. One suggested analytic method is to perform mediation analysis to measure the direct, indirect, and mediation effects. With respect to the path analysis, future research could include a comparison of the efficacy of caregiver-friendly workplace programs by employee group, faculty, or amongst a larger sample of carer-employees. For example, it would be useful to know if there are differences in the workplace experience between faculties. Doing such comparative analysis would require a larger response rate. As mentioned earlier, there are approximately 7000 employees in the public post-secondary institution of concern, with both surveys capturing between 10–12% of the entire community. A higher response rate would lead to a reduction in data skewness, and more accurate descriptive statistics and path analyses. Further, the general ability of the results is limited given that a single post-secondary institution has been sampled. Another limitation is confronting the endogeneity issue in the SEM model. The efficacy of caregiver-friendly workplace programs is one causal effect to changes in workplace experience; however, there may be other causal effects. For example, workplace experience could be caused by the nature of work conducted by the employee, the type of environmental setting (e.g., plenty of natural lighting and space), and the amount of work flexibility. If feasible, these variables would be useful to collect for future studies; otherwise, an alternative is to adopt the instrument variables (IV) or use two-stage least squares methods (2SLS).

## Conclusion

This study examines the effectiveness of caregiver-friendly workplace program over time, particularly changes in the workplace culture through workplace experience. Implementation and awareness of caregiver friendly workplace programs was found to indirectly impact the workplace experience in a positive manner. More importantly, this study advocates for positive changes in the workplace experience by improving employees’, and specifically carer-employees’ work-life balance; thus, having a more positive workplace culture. This study concludes that the awareness of caregiver-friendly workplace programs positively impacts the amount of support received, which in turn augments the workplace experience. From this perspective, the implementation of caregiver-friendly workplace programs appears to be effective. The efficacy of caregiver-friendly workplace programs likely varies, depending on the actions of support stakeholders. Therefore, the results regarding to the changes over time suggest the efficacy of caregiver-friendly workplace programs may need to be reassessed belatedly, due to the lag in improved relationships between support stakeholders (i.e., senior management) and employees. Overall, results suggest the guidelines for organizational changes be incorporated for both carer-employees and non-carer-employees, to improve work-life balance while retaining productivity and talent.

## Supporting information

S1 Table Appendix A. Cross-tab of employment and caregiving statistics between T1 and T2.
(DOCX)
