# Supplementary material for: Evaluating changes in workplace culture: Effectiveness of a caregiver-friendly workplace program in a public post-secondary educational institution
Source: PLoS One. 2021 May 14;16(5):e0250978. doi: 10.1371/journal.pone.0250978 (PMC8121345; doi:10.1371/journal.pone.0250978)
Supplement: S1 Table — Cross-tab of employment and caregiving statistics between T1 and T2. (DOCX) [file pone.0250978.s001.docx]

**Appendix A –** Cross-tab of employment and caregiving statistics between T1 and T2

| **Variable** | **Value** | **T1 (n = 747)** | **T2 (n = 816)** |
| --- | --- | --- | --- |
| ***Employment Related*** | | | |
| *Faculty* | Arts & Science | 0.0 | 0.1 |
|  | Business | 3.2 | 5.4 |
|  | Engineering | 4.6 | 3.9 |
|  | Health Sciences | 43.1 | 43.8 |
|  | Humanities | 2.1 | 4.2 |
|  | Science | 8.7 | 6.9 |
|  | Social Sciences | 5.5 | 5.3 |
|  | Not Applicable | 32.8 | 30.4 |
| *Employee Group* | Academic | 11.1 | 20.1 |
|  | Facilities & Services | 0.8 | 0.6 |
|  | Bargaining Units | 86.2 | 78.7 |
|  | Non-Bargaining Units | 1.9 | 0.5 |
| *Hours of Employment* | Full-Time (30+) | 91.8 | 92.3 |
|  | Part-Time | 7.4 | 6.6 |
|  | Other | 0.8 | 1.1 |
| *Employment Status* | Continuing | 80.5 | 89.0 |
|  | Contract | 0.0 | 0.0 |
|  | Limited Term | 13.0 | 9.3 |
|  | Other | 6.5 | 1.6 |
| *Supervisory Role* | Yes | 27.2 | 35.5 |
|  | No | 72.8 | 64.5 |

| ***Caregiving Variable*** | **Value** | **T1** | **T2** |
| --- | --- | --- | --- |
| *Caregiver-Employee* | Yes | 14.6 | 19.7 |
| *Number of Care-Recipients* | N/A or 0 | 85.4 | 80.3 |
|  | 1 | 9.4 | 12.5 |
|  | 2 | 3.9 | 5.1 |
|  | 3+ | 0.7 | 1.0 |
| *Age of Care Recipient (Primary)* | N/A | 85.4 | 80.3 |
|  | 40 yrs. or less | 0.9 | 2.0 |
|  | 41-60 | 1.3 | 2.4 |
|  | 61+ | 11.7 | 14.3 |
| *Relationship to Care-Recipient* | N/A | 85.4 | 80.3 |
|  | Immediate Family | 13.5 | 17.4 |
|  | Extended Family | 0.1 | 0.2 |
|  | Other | 0.3 | 1.0 |
| *Live with Care Recipient* | N/A | 85.4 | 80.3 |
|  | Yes | 5.2 | 5.2 |
|  | No | 8.8 | 13.6 |
| *Received Assistance with Caregiving* | N/A | 85.4 | 80.3 |
|  | Yes | 10.0 | 13.5 |
|  | No | 4.6 | 6.2 |
| *Get Paid for Caregiving* | N/A | 85.4 | 80.3 |
|  | Yes | 0.0 | 0.1 |
|  | No | 14.6 | 19.6 |
| *Weekly Hrs. of Caregiving* | N/A | 85.4 | 80.3 |
|  | 0 – 9 hrs. | 8.9 | 11.7 |
|  | 10 – 19 hrs. | 3.0 | 5.2 |
|  | 20+ hrs. | 2.0 | 1.7 |
